# Sensing Protein Structural Transitions with Microfluidic Modulation Infrared Spectroscopy

**DOI:** 10.3390/bios15060382

**Published:** 2025-06-13

**Authors:** Lathan Lucas, Phoebe S. Tsoi, Ananya Nair, Allan Chris M. Ferreon, Josephine C. Ferreon

**Affiliations:** 1 Department of Biochemistry and Molecular Pharmacology, Baylor College of Medicine, Houston, TX 77030, USA; lathanl@bcm.edu (L.L.); phoebe.tsoi@bcm.edu (P.S.T.); an107@rice.edu (A.N.); 2 Department of Biosciences, Rice University, Houston, TX 77005, USA

**Keywords:** microfluidics, infrared spectroscopy, aggregation, protein secondary structure, structural transitions, tau, intrinsically disordered proteins

## Abstract

Microfluidic modulation spectroscopy-infrared (MMS) offers a label-free, high-sensitivity approach for quantifying changes in protein secondary structures under native solution conditions. MMS subtracts the solvent backgrounds from sample signals by alternately flowing proteins and matched buffers through a microfluidic chamber, yielding clear amide I spectra from microliter volumes. In this study, we validated MMS on canonical globular proteins, bovine serum albumin, mCherry, and lysozyme, demonstrating accurate detection and resolution of α-helix, β-sheet, and mixed-fold structures. Applying MMS to the intrinsically disordered protein Tau, we detected environment-driven shifts in transient conformers: both the acidic (pH 2.5) and alkaline (pH 10) conditions increased the turn/unordered structures and decreased the α-helix content relative to the neutral pH, highlighting the charge-mediated destabilization of the labile motifs. Hyperphosphorylation of Tau yielded a modest decrease in the α-helical fraction and an increase in the turn/unordered structures. Comparison of monomeric and aggregated hyperphosphorylated Tau revealed a dramatic gain in β-sheet and a loss in turn/unordered structures upon amyloid fibril formation, confirming MMS’s ability to distinguish disordered monomers from amyloids. These findings establish MMS as a robust platform for detecting protein secondary structures and monitoring aggregation pathways in both folded and disordered systems. The sensitive detection of structural transitions offers opportunities for probing misfolding mechanisms and advancing our understanding of aggregation-related diseases.

## 1. Introduction

The quantitative characterization of structurally heterogeneous protein ensembles under diverse conditions remains a central challenge in molecular biophysics [1]. Proper folding underlies protein function, whereas misfolding and aberrant aggregation frequently drive pathology [2,3,4,5,6]. Intrinsically disordered proteins (IDPs), which lack stable structures, further complicate this landscape by also sampling dynamic ensembles of transient α-helices, β-sheets, and turns [7,8,9,10,11,12,13]. Capturing these transient conformers and their trajectories toward ordered aggregates requires spectroscopic techniques with high sensitivity, rapid acquisition, and effective suppression of solvent backgrounds.

Conventional structural detection techniques exhibit critical limitations in detecting IDP structures [14]. Circular dichroism (CD) reports global helical content but exhibits difficulty in resolving overlapping structural contributions or detecting weak β-sheet signals in complex mixtures unless optimized specifically for IDP analysis [15,16,17,18,19]. Fourier transform infrared spectroscopy (FTIR) monitors peptide-backbone vibrations but is compromised by strong H₂O absorbance and therefore necessitates deuterated solvents, which can limit the buffer compatibility for studying protein behavior across various conditions [20,21,22]. Nuclear magnetic resonance yields residue-specific information but demands high protein concentrations and becomes impractical for studying large proteins or fast kinetics [23,24,25,26]. X-ray crystallography and cryo-electron microscopy are techniques that require a minimal number of alternate structures or depend on averaging data from well-represented structural states, respectively, and are inherently limited in their ability to assess disordered ensembles in solutions [27,28,29,30,31]. Therefore, recent efforts have focused on developing and refining high-resolution methodologies capable of resolving the fleeting conformers populated by IDPs.

Microfluidic modulation spectroscopy-infrared (MMS) utilizes an alternating-flow design that delivers back-to-back protein and buffer segments through a microfluidic chamber [32,33]. Mechanical valves switch the flow between the sample and a matched buffer, “blank”, and real-time subtraction of sequential spectra accounts for solvent absorbance without the need for deuteration. Repeated modulation cycles amplify genuine protein signals, improving the signal-to-noise ratio over conventional single-pass measurements. Microliter-scale sample volumes and sub-minute acquisitions enable the analysis of scarce or labile proteins as well as the time-resolved monitoring of transient structures, conformational transitions, and aggregation kinetics [33].

MMS is one of several solution-phase techniques—alongside single-molecule Förster resonance energy transfer (smFRET), fluorescence-lifetime imaging microscopy (FLIM), hydrogen–deuterium exchange mass spectrometry (HDX-MS), small-angle X-ray scattering (SAXS), and CD—that have advanced our ability to monitor protein structural shifts and IDP aggregation [32,34,35,36,37,38,39,40,41,42,43]. However, MMS uniquely offers the detection of intrinsic label-free protein signals and solvent background subtraction to yield real-time, high-fidelity extraction of the amide I signature in pure H_2_O [44]. Unlike optical methods that depend on extrinsic probes (smFRET, FLIM), IR directly measures a protein’s backbone absorption, yielding an immediate, quantitative readout of secondary-structure content [44]. The workflow requires only microliter sample volumes, with no deuteration or dilution, yet achieves sensitivity sufficient to detect subtle helix–sheet transitions that often evade CD or conventional IR techniques [45,46]. While smFRET, FLIM, HDX-MS, and SAXS each interrogate specific aspects of structures, typically at the cost of specialized labeling, complex sample preparation, or limited throughput, MMS delivers rapid, quantitative secondary-structure fingerprints with minimal perturbation [33,47].

Methodologically most similar to MMS is attenuated-total-reflection IR (ATR-FTIR), which can report amide I features but remains constrained by bulk solvent interference, single-pass acquisition, and limitations in background subtraction [20,48]. In contrast, MMS employs alternating flows of protein samples and matched buffers, combined with rapid quantum-cascade laser interrogation, to achieve label-free secondary-structure quantification with sub-minute temporal resolution and high signal-to-noise ratios. MMS requires only microliter volumes of aqueous protein and no deuteration, yet can discern subtle α-helix to β-sheet shifts and detect early β-rich aggregation with far greater sensitivity than single-pass ATR-FTIR. Moreover, MMS’s automation and 96-well compatibility enable high-throughput screening of conditions and time courses [33]. By filling the niche for rapid, quantitative secondary-structure analysis in scarce, dynamic, or aggregation-prone proteins such as intrinsically disordered Tau, MMS provides an indispensable complement to established biophysical methods [49].

In this study, we first validated MMS against canonical folded proteins, demonstrating secondary-structure proportions that agreed with sub-2.5 Å X-ray reference values. We then applied MMS to the IDP Tau, detecting pH-dependent shifts in its conformers and monitoring structural transitions induced by hyperphosphorylation. Finally, we distinguished monomeric pTau from its β-rich amyloid aggregates. These results established MMS as a robust, label-free platform for quantitative secondary-structure analysis in both folded and disordered systems. These techniques’ compatibility with native buffers, minimal sample requirements, and rapid acquisition make MMS ideally suited for probing dynamic ensembles, screening modulators of disorder, and mapping aggregation pathways in neurodegenerative and other protein conformation disorders.

## 2. Materials and Methods

### 2.1. Modeling of ⍺-Helical and β-Sheet Proteins

Model spectra representing α-helix and β-sheet secondary structures were generated in Excel by assigning an intensity value of 1.0 to wavenumbers associated with predicted α-helical or β-sheet features, while all other wavenumbers were constrained to values of ≤0.1 to simulate background signals. These synthetic spectra were subsequently smoothed using a 19-point Savitzky–Golay filter to reduce noise and approximate realistic peak profiles [50]. Following smoothing, the second-derivative spectra and their inversions were calculated to facilitate comparison with the experimental datasets.

### 2.2. Purification of Recombinant Tau and pTau Proteins

Wild-type (WT) Tau and hyperphosphorylated Tau (pTau) were overexpressed in *E. coli* BL21 star cells and cultured at 37 °C in Terrific Broth medium containing carbenicillin. Protein expression was induced by the addition of 1 mM isopropyl β-d-1-thiogalactopyranoside (IPTG), and the cells were grown overnight at 18 °C. The cells were centrifuged at 4000× *g*, and the pelleted cells were resuspended in 50 mM NaCl, 5 mM DTT, and 50 mM sodium phosphate at pH 6.5 and protease inhibitor cocktail (GeneDEPOT, Houston, TX, USA). The cells were homogenized and subsequently centrifuged at 50,000× g for 30 min. The supernatant was adjusted to a final NaCl concentration of 450 mM and incubated in near-boiling water (~85–95 °C) for 30 min. Following heat treatment, the lysate was centrifuged once more and diluted to 50 mM NaCl. The supernatant was then added to a heparin Sepharose HP (Cytiva, Marlborough, MA, USA) column for FPLC purification and eluted by a salt gradient of 0–600 mM NaCl. The fractions containing Tau were harvested, concentrated, and further purified through reverse-phase HPLC using a C18 column (Agilent, Santa Clara, CA, USA). The purified Tau was aliquoted, lyophilized, and stored at −80 °C for later use. The protein integrity and purity were evaluated through SDS-PAGE followed by Coomassie Blue staining.

### 2.3. Sample Preparation

Stock solutions: Recombinant bovine serum albumin (BSA; GeneDEPOT, Houston, TX) and egg-white lysozyme (Amresco, Framingham, MA, USA) were purchased. Recombinant mCherry was purified by Superdex 200 (Cytiva) from the cleavage product yielded from a previous study [51]. Full-length human Tau 2N4R (WT Tau) and hyperphosphorylated Tau (pTau) were purified as described above.

Protein quantification: Concentrations were determined spectrophotometrically by the Edelhoch method, which exploits the intrinsic absorbances of Trp and Tyr residues at 280 nm [52,53,54]. Briefly, aliquots were denatured in 6 M guanidinium hydrochloride, and absorbance was measured using a NanoDrop 2000C Spectrophotometer (Thermo Fisher, Waltham, MA, USA). Concentrations were calculated from the Beer–Lambert law and adjusted to 1 or 2 mg/mL (BSA, Lysozyme, and mCherry) or 0.1 mg/mL (WT Tau and pTau) for the MMS analyses.

Buffer conditions: For the MMS analysis, mCherry was diluted in 20 mM sodium phosphate and 100 mM NaCl at pH 7.1. BSA and lysozyme were diluted in ultrapure water (18.2 MΩ cm). WT Tau and pTau were diluted in 10 mM phosphate buffers (Thermo Scientific; pH 7, 2.5, or 10). Identical buffer conditions were used for the buffer matching for each protein during the MMS runs to ensure that no variation in the spectra occurred due to changes in the ion or solute concentrations across the various buffer conditions.

### 2.4. Microfluidic Modulation Spectroscopy (MMS)

Secondary-structure characterization of the standardized protein samples was performed using the Aurora RedShiftBio Microfluidic Modulation Spectroscopy (MMS) system (RedShiftBio, Boxborough, MA, USA), collecting successive spectra; the real-time subtraction of the buffer trace eliminated solvent absorbance and baseline drift, yielding a difference spectrum that reported exclusively on the protein backbone vibrations. The samples were organized into two groups: (1) mCherry and its corresponding matched buffer and (2) BSA and lysozyme, analyzed independently but matched to corresponding ultrapure water buffer conditions. Each sample was loaded into a 12 × 8 well plate positioned adjacently to its corresponding buffer as per the MMS system guidelines. Each sample was loaded at 1 mg/mL into a 96-well clear round-bottom plate (Thermo Scientific, Waltham, MA, USA). Measurements were conducted using the “highly transparent buffer” setting enabled to extend the detector’s intensity cut-off. Each sample and its corresponding buffer were analyzed over a ~10 min runtime. The Tau and pTau were run similarly using 0.1 mg/mL of each protein matched to a phosphate buffer of either pH 7, 10, or 2.5.

For measuring the thermal denaturation of lysozyme (2 mg/mL), the samples were run in triplicate for an extended run time of ~2 h with a temperature ramp of 1 °C/min. In total, ~25 spectral reads were performed at 25 °C and another ~25 reads were performed at 95 °C. The spectra for each of the triplicate were averaged for simplified analysis.

To generate amyloid aggregates, pTau (50 µM) was incubated with 5 µM heparin sulfate (Sigma-Aldrich) in ultrapure water at 37 °C for 48 h. Fibril formation was confirmed at various time points by Thioflavin-T fluorescence (ThT) microscopy, where samples were mixed in triplicate with 3 µM ThT and placed on a sealed coverslip for fluorescence imaging and intensity analysis using the EVOS FL Imaging System (Invitrogen) and Fiji (ImageJ 2.9.0), respectively. Samples at various time points (0–48 h) were probe-sonicated on ice (three 5 s bursts at 20 kHz, 30 s cooling intervals) and spun down at 15,000× *g* for 30 min at 4 °C. The supernatants were immediately analyzed via MMS to monitor the aggregate formation with time. At the 48 h time point, the aggregates were pelleted at 15,000× *g* for 30 min at 4 °C; the supernatant was discarded; and each pellet was washed once with H_2_O, resuspended to the original volume, and probe-sonicated on ice (three 5 s bursts at 20 kHz, 30 s cooling intervals) immediately before MMS measurement. The concentration of the pTau aggregates (~1.5 µM or ~3% of the total original pTau) isolated from each pellet was determined using UV-Vis, as described above.

### 2.5. IR Spectrum Analysis

The differential absorbance spectra were processed using Delta Analytics software version 2.10.126.133 (RedshiftBio). The spectra were analyzed with a 0.6 nominal displacement factor and fit over a custom range of 1600 to 1700 cm^−^^1^ to a lysozyme model. The protein concentration fit and protein displacement factor were set to 30% and 20%, respectively, as recommended by RedshiftBio, to account for protein space occupancy. Savitzky–Golay smoothing was applied to the second-derivative plots generated for all samples with a 19 wavenumber window [50]. Both ends of the second-derivative spectra were baselined. The inversions of the baseline-corrected second-derivative spectra were then fit to Gaussian curves. The areas of the Gaussian curves were used to estimate the percentages of higher-order structures.

### 2.6. Statistical Analysis

Unless otherwise stated in the figure legends, T-test comparisons were employed for the direct comparisons between the samples. All data points (MMS spectra) were collected in three independent replicates. All MMS spectra show the standard error of the mean (SEM) shaded above and below the average. All bar graphs show the average and SEM. Regardless of the analysis method, significance levels are denoted by *, **, ***, and **** for *p* < 0.05, *p* < 0.01, *p* < 0.001, and *p* < 0.0001. The graphs were generated using Prism v. 10.3.0 (GraphPad, San Diego, CA, USA).

## 3. Results

### 3.1. Microfluidic Modulation Spectroscopy-Infrared (MMS) Enables Quantitative Analysis of Protein Secondary Structure

**Figure 1:**
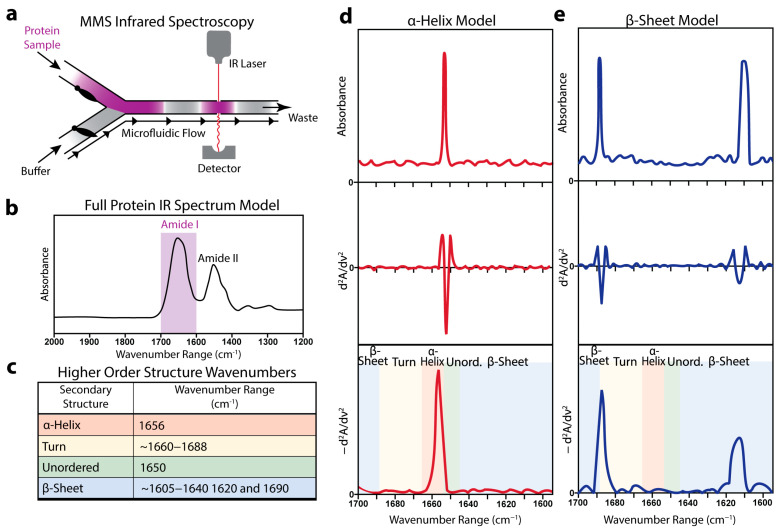
Microfluidic modulation infrared spectroscopy can detect protein secondary structures. (**a**) Schematic representation of the microfluidic flow chamber showing the back-to-back mechanical modulation of a protein sample and a matched buffer flowing through the IR laser and detector. (**b**) Representative full IR spectrum of a typical protein with the amide I spectral peak highlighted in purple [60,61]. (**c**) Table displaying experimentally determined IR wavenumber absorbance peaks that correspond to protein secondary structures [33,57,58,59,60,61]. (**d**) Representative modeled MMS spectra of a pure α-helical protein. The top panel shows the raw absorbance values, the middle panel shows the second derivative of the raw absorbance values, and the bottom panel shows the baseline-corrected inverted second-derivative spectra used for secondary structure analysis. (**e**) Similar representative modeled MMS spectra of pure β-sheet protein.

MMS integrates an alternating-flow design in which mechanical valves cycle between a protein solution and a matched buffer, delivering successive segments through a fixed infrared beam path (Figure 1a). As each segment traverses the detector, real-time subtraction of the buffer absorbance from the sample reads isolates the protein signal. The resulting spectrum displayed a dominant amide I envelope between 1600 and 1700 cm^−1^ that arose from the C=O stretching vibrations of the peptide backbone sensitive to the secondary-structure variations (Figure 1b) [55,56].

The assignments of the principal sub-bands to ⍺-helix, β-sheet, turn, and unordered motifs are summarized in the reference table (Figure 1c) [33,57,58,59]. To sharpen the overlapping features, the absorbance profiles underwent baseline correction and inverted second-derivative processing, followed by normalization to the unit integral. This derivative analysis enhanced the spectral resolution and enabled motif-specific peak identification independent of absolute absorbance intensity.

The modeled raw amide I sub-bands for a purely α-helical protein and a β-sheet protein exhibited distinct peak shapes and positions (Figure 1d,e). Their corresponding derivative MMS spectra revealed well-resolved peaks at motif-specific wavenumbers, facilitating a direct comparison of the structural populations across the samples.

### 3.2. Validation of MMS-Based Secondary-Structure Analysis Using Model Proteins

**Figure 2:**
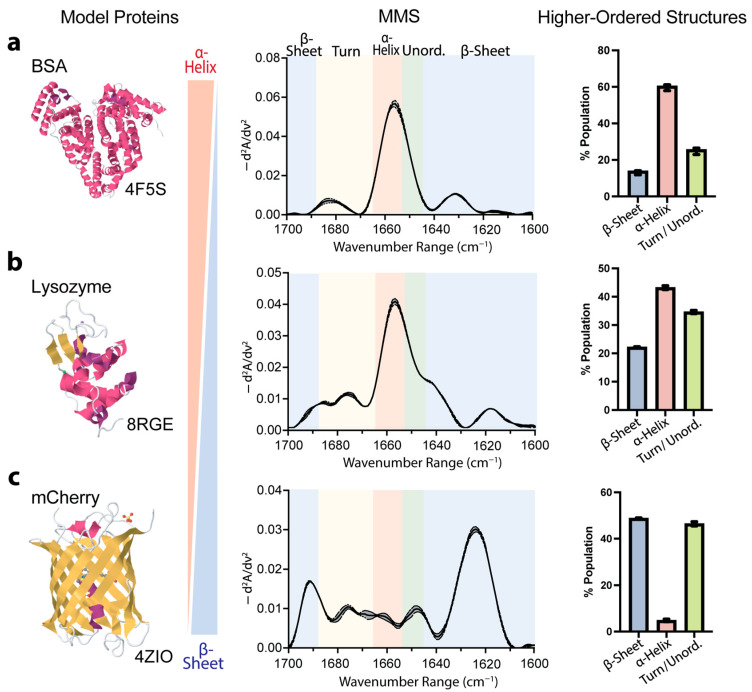
MMS quantitatively distinguishes between proteins exhibiting different secondary-structure compositions. (**a**) Previously published X-ray crystal structure [62], MMS spectrum, and higher-order structures predicted by Gaussian curve fitting of primarily α-helical protein bovine serum albumin (BSA). n = 3, error bars = SEM. (**b**) Previously published X-ray crystal structure [63], MMS spectrum, and higher-order structures predicted by Gaussian curve fitting of mixed α-helical/β-sheet protein lysozyme. n = 3, error bars = SEM. (**c**) Previously published X-ray crystal structure [64], MMS spectrum, and higher-order structures predicted by Gaussian curve fitting of primarily β-sheet fluorescent protein mCherry. n = 3, error bars = SEM. For all crystal structures, α-helices are colored in pink, β-sheets are colored in yellow, and both turns and disordered are in white.

To validate the accuracy and capability of MMS for secondary-structure determination, we analyzed three well-characterized model proteins: bovine serum albumin (BSA), lysozyme, and the fluorescent protein mCherry. These proteins represent distinct structural compositions, predominantly α-helical, mixed α/β structures, and predominantly β-sheet, respectively, providing robust benchmarks for assessing the technique’s reliability. The MMS analysis of the BSA revealed a spectral signature consistent with its known crystal structure [62], exhibiting a ~60% α-helical structure (Figure 2a). The lysozyme, which exhibited mixed α-helix and β-sheet features, showed a heterogeneous secondary-structure composition, with MMS-derived values (~43% α-helical, ~22% β-sheet) in close agreement with its crystal structure [63] (Figure 2b). Similarly, the mCherry exhibited spectral features characteristic of β-sheet structures, with the MMS-derived values indicating a ~50% β-sheet composition (Figure 2c) [64].

These results confirm MMS as a highly accurate and reproducible method for structural analysis. This technique effectively distinguishes between different structural profiles and quantifies complex secondary-structure compositions with precision comparable to high-resolution crystallography. Its ability to accurately assess diverse structural elements, from predominantly α-helical to predominantly β-sheet proteins, demonstrates its broad applicability for structural characterization across various protein classes.

### 3.3. Protein Thermal Unfolding Can Be Monitored by MMS

**Figure 3:**
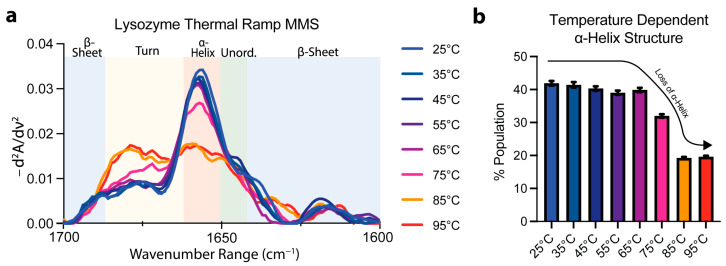
MMS detects structural transitions during lysozyme thermal denaturation. (**a**) MMS inverted second-derivative spectra of lysozyme (2 mg/mL, averaged at each time point for n = 3), indicating that most of the folded-to-unfolded structural transitions occur between 65 °C and 85 °C. (**b**) Gaussian curve fitting of the α-helical populations found within lysozyme at each temperature during thermal denaturation. n = 3, error bars = SEM.

To confirm the specificity of the MMS spectra to secondary-structure detection, lysozyme MMS spectra were obtained as a function of temperature using a thermal ramping continuum from 25 °C to 95 °C. The spectra obtained at temperatures below ~75 °C indicated a general conservation of the a ⍺-helix peak at ~1654–1656 cm^−1^ (Figure 3a). At 75 °C, the ⍺-helix peak became drastically reduced with a marked increase in turn identity noted by the peak formation at ~1680–1681 cm^−1^ (Figure 3a). After performing of Gaussian curve fitting to quantify the secondary-structure abundance, the ⍺-helix identity was reduced to ~18% from ~42% in the lysozyme during the structural transition linked to thermal denaturation (Figure 3b). These results confirmed the specificity of MMS in secondary-structural detection and suggest that MMS is well-suited to studying protein structural transitions. A more detailed thermodynamic analysis of MMS data will yield stability parameters like transition midpoints and enthalpies of unfolding [42,54].

### 3.4. Tau Exhibits Secondary-Structure Changes Under Variable pH

**Figure 4:**
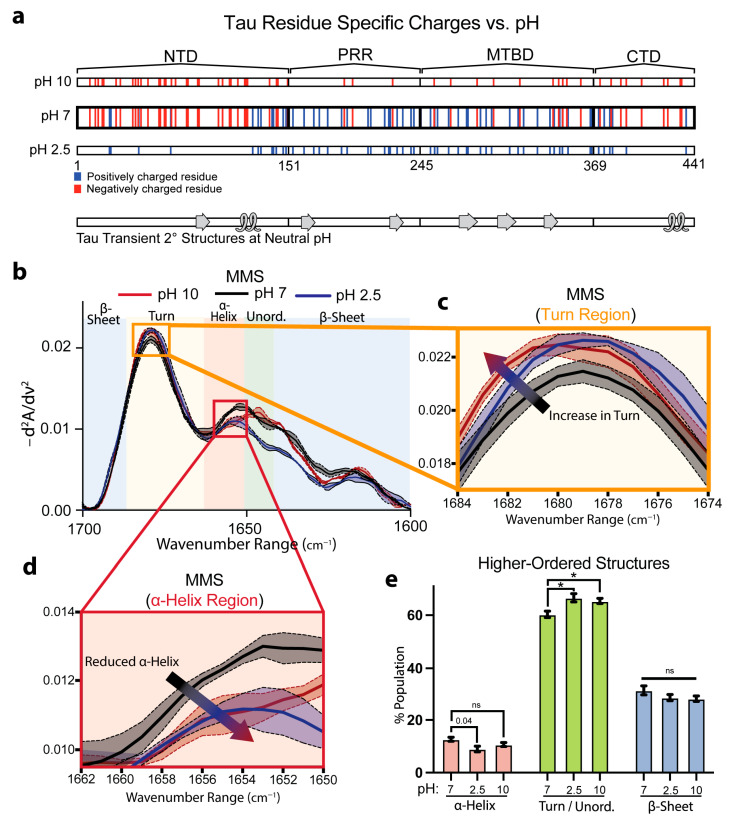
MMS detects subtle pH-dependent changes in Tau secondary structures. (**a**) Charges of residues along full-length 2N4R Tau across various pH values with native transient secondary-structure elements highlighted (β-sheet = arrow, α-helix = loop) [65]. Tau consists of an N-terminal domain (NTD), a proline-rich region (PRR), a microtubule binding domain (MTBD), and a C-terminal domain (CTD). (**b**) MMS inverted second-derivative spectra of Tau measured in solutions of various pH values. n = 3, shaded regions = SEM. (**c**) Inset of panel b highlighting the turn region of the MMS spectra. The arrow indicates the peak shift trend. (**d**) Inset of b highlighting the α-helical region of the MMS spectra. The arrow indicates the peak shift trend. (**e**) Gaussian curve fitting of the inverted second-derivative spectra, indicating the abundances of α-helix, turn/unordered, and β-sheet populations across the various sampled conditions. n = 3; error bars = SEM; * = *p* < 0.05; ns = not significant.

We next evaluated the capacity for MMS to detect pH-dependent shifts in full-length Tau. Prior solution NMR studies on native Tau structures have mapped out labile α-helical and β-sheet motifs stabilized in part by electrostatic interactions among charged side chains at pH 6.9 (Figure 4a) [65]. We therefore compared the Tau spectra in neutral (pH 7), acidic (pH 2.5), and alkaline (pH 10) conditions, which altered the protonation states of the ionizable residues. Acidification to pH 2.5 protonated the Asp and Glu side chains, neutralizing negative charges, while alkalinization toward pH 10 partially deprotonated the Lys, Arg, and Cys residues and began to deprotonate the Tyr phenols, reducing the overall positive charge (Figure 3a).

Inverted, normalized second-derivative MMS spectra revealed that at pH 2.5, the signals between the wavenumbers of ~1650 and 1620 cm^−1^ drastically diverged from the pH 7 and 2.5 conditions (Figure 4b). However, both extreme pH conditions exhibited modest attenuation of the β-sheet minima, with a more pronounced peak shift at the turn region from 1679 cm^−1^ at pH 7 to 1681 cm^−1^ at pH 2.5 and 10. Furthermore, there was a peak reduction at ~1640 cm^−1^ at pH 2.5 in the unordered region (Figure 4b,c). Interestingly, the MMS spectra showed reduced ⍺-helix formation with a wavenumber shift from 1656–1654 cm^−1^ at pH 7 to 1654–1648 cm^−1^ at pH 2.5 and 10 (Figure 4d), suggesting a charge dependency for helical formation in Tau. Gaussian fitting of the IR spectra to determine the secondary-structure populations corroborated these observations: compared with pH 7, the Tau at pH 2.5 showed reductions in the transient ⍺-helix structures and, at pH 2.5 and 10, exhibited increases in turn/unordered fractions (Figure 4e). The loss of structure at the acidic pH matches previous studies suggesting acidic conditions reduce Tau folding and compaction [66]. These structural changes are notably subtle and reflect the nuanced differences between the various pH-dependent Tau ensembles, underscoring the high sensitivity of MMS.

### 3.5. Hyperphosphorylation Moderately Affects Tau Secondary Structures

**Figure 5:**
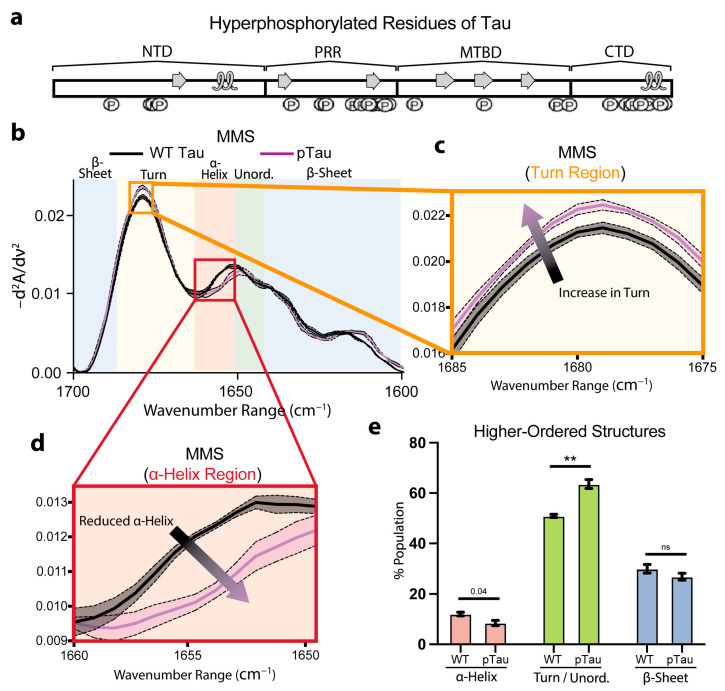
Hyperphosphorylation of Tau moderately affects pre-fibrillar native secondary structures. (**a**) Phosphorylation sites of residues along full-length 2N4R Tau, with native transient secondary-structure elements labeled (β-sheet = arrow, α-helix = loop) [65,67]. (**b**) MMS inverted second-derivative spectra of WT Tau and pTau. n = 3, shaded regions = SEM. (**c**) Inset of panel b highlighting the turn region of the MMS spectra. The arrow indicates the peak shift trends. (**d**) Inset of b highlighting the α-helical region of the MMS spectra. The arrow indicates the peak shift trends. (**e**) Gaussian curve fitting of the inverted second-derivative spectra indicating the abundance of α-helix, turn/unordered, and β-sheet populations across the various sampled conditions. n = 3, error bars = SEM, ** = *p* < 0.01, ns = not significant.

To investigate how phosphorylation alters Tau secondary structures, we compared wild-type (WT) Tau with hyperphosphorylated Tau (pTau). We previously mapped the Ser, Thr, and Tyr residues modified in our recombinant pTau preparation, highlighting sites within the known transient C-terminal α-helix (Figure 5a) [67].

The inverted, normalized second-derivative spectra overlaid for the WT Tau and pTau (Figure 5b) revealed largely congruent spectra that retained the hallmark minima and maxima of α-helix, β-sheet, and turn/unordered motifs. However, the pTau exhibited a discernible increase in the turn features (Figure 5c) and a notable reduction in the α-helix features (Figure 5d), consistent with disruption of the C-terminal helix by phosphate incorporation at residues involved in α-helix formation (Figure 5a,d). Furthermore, the Gaussian fitting of the spectra to quantify the secondary-structure populations indicated a nearly significant (*p* = 0.04) decrease in the α-helical fraction for the pTau relative to the WT (Figure 5e). Concurrently, we observed a significant increase in the turn/unordered content in pTau (Figure 5e). These results show that Tau phosphorylation moderately perturbs local folding propensities, yet the global conformational ensemble of monomeric pTau remained largely preserved. Even though extensive studies have shown that hyperphosphorylation ultimately drives larger structural rearrangements and fibril formation [68,69], we monitored the secondary-structure perturbations of the pTau prior to any self-association. Consequently, MMS provides a highly sensitive platform for quantifying the earliest pre-aggregative effects of post-translational modifications on intramolecular disorder and dynamic secondary structures in intrinsically disordered proteins.

### 3.6. MMS Detects pTau Aggregation

**Figure 6:**
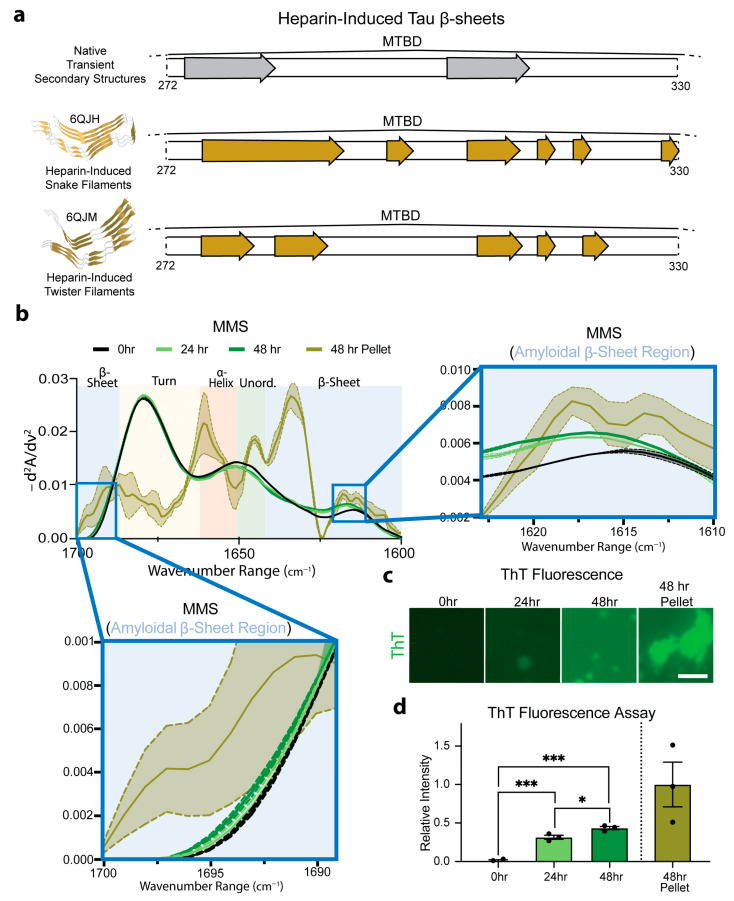
MMS monitors pTau aggregation in real time. (**a**) Schematic representation of the MTBD regions of native transient β-sheet structures, highlighted with gray arrows, and of stable β-sheets found in heparin-induced aggregates, highlighted with yellow arrows [55,61]. (**b**) MMS inverted second-derivative spectra of monomeric pTau and heparin-induced aggregated pTau. The high-intensity peak for the amyloidal β-sheet region is shown in the right inset and the low-intensity peak shown in the bottom left inset [71]. n = 3, shaded regions = SEM. (**c**) Representative fluorescence microscopy of 3 µM ThT-stained samples of 50 µM Tau incubated with 5 µM heparin sulfate imaged after 0, 24, and 48 h. After 48 h, the samples were pelleted to isolate amyloids for ThT staining. Scale bar = 10 µm. (**d**) Plotted relative ThT intensity per pixel derived from fluorescence microscopy images. n = 3, error bars = SEM, * = *p* < 0.05, *** = *p* < 0.001.

To evaluate the ability to use MMS to distinguish monomeric and aggregated forms of hyperphosphorylated Tau (pTau), we induced aggregation by incubating the pTau with heparin sulfate at 37 °C and measured the MMS absorbance spectra of the aggregates. The incubation of Tau with heparin has previously been shown to induce the formation of amyloid protein snake filaments and twister filaments (Figure 6a) [70]. The β-sheets in native Tau structures do not entirely overlap with those adopted upon aggregation, suggesting substantial structural shift during heparin-induced amyloidogenesis. This structural shift was measured in real time and compared to isolated aggregates (Figure 6a). The control measurements confirmed that the heparin sulfate alone produced no detectable signal under our conditions.

The MMS inverted, normalized second-derivative spectra collected for the pTau (50 µM) incubated with heparin sulfate (5 µM) for 0, 24, and 45 h showed a general progression toward increased amyloid β-sheet signatures between ~1630–1611 and ~1685–1695 cm^−1^ (Figure 6b) [71]. After 48 h, the samples were pelleted to isolate a pure population of amyloid aggregates (~1.5 µM; approximately 3% of the total pTau in the solution) and analyzed using MMS. These isolated aggregates exhibited pronounced maxima in the β-sheet regions, which were absent or minimal in the monomeric pTau MMS spectrum (Figure 6b). These marked spectral shifts reflect the formation of tightly packed, hydrogen-bonded β-sheets characteristic of amyloid fibrils previously shown to form amyloid snake filaments and twister filaments in the presence of heparin [70] (Figure 6a). Thioflavin-T staining was used to confirm the molecular progression of the amyloid formation in each sample (Figure 6c), indicating a significant but moderate time-dependent increase in the amyloid formation from 0 to 48 h (Figure 6d), which was detected by MMS in real time (Figure 6b). The loss of flexible turns apparent in the 48 h pelleted isolated amyloids alongside the gain of the rigid β-sheets indicates the possible conversion of transient turns into stable cross-β architectures during fibril formation.

These results demonstrate that MMS can sensitively detect and quantify the transition from largely disordered monomers to ordered amyloid fibrils. The pronounced change in the β-sheet signals underscores MMS’s utility for real-time monitoring of aggregation kinetics and for distinguishing distinct structural states in protein misfolding.

## 4. Discussion

The present study establishes microfluidic modulation spectroscopy-infrared (MMS) as a rigorous solution-phase approach for dissecting protein secondary structures across the structural continuum from ordered globular folds to highly dynamic intrinsically disordered ensembles. Benchmarking against BSA, mCherry, and lysozyme confirmed that MMS accurately reproduces canonical α-helix, β-sheet, and mixed-fold signatures in native aqueous buffers, obviating the need for D₂O substitution and validating this method’s compatibility with various buffer conditions. The results position MMS as an immediately deployable alternative to conventional FTIR and CD for rapid, label-free secondary-structure quantification.

The application to the Tau revealed that electrostatic perturbations reshape dynamic conformer populations. The protonation of the Asp and Glu residues at pH 2.5 and the deprotonation of the Lys, Arg, Cys, and Tyr residues at pH 10 produced subtle yet reproducible shifts in the α-helix turn/unordered content, whereas the overall β-sheet fraction remained comparable with that observed at the neutral pH. These findings align with reports that extreme pH conditions attenuate Tau aggregation [66], suggesting that the perturbation of transient elements may increase conformational flexibility and reduce the propensity to nucleate pathogenic assemblies. Furthermore, the pronounced loss of the α-helical signal and the concomitant increase in the unordered content we observed at pH 2.5 mirror a previous demonstration that mildly acidic conditions (pH 4) induce the full unfolding of Tau and eliminate its compact subpopulations [66]. In the cellular context, late endosomes and lysosomes maintain acidic pH values of ~4.5–5.5; under these acidic conditions, Tau may lose secondary structures, prior to proteolytic processing or aggregation [72]. Such acid-induced unfolding may expose normally buried segments of Tau, facilitating cleavage by lysosomal proteases into amyloidogenic fragments. By recapitulating these pH-driven structural transitions in vitro, our MMS data directly link Tau’s conformational plasticity under acidic conditions to potential pathological processing and aggregation in Alzheimer’s disease.

The hyperphosphorylation elicited a parallel spectral response; the incorporation of the negatively charged phosphate groups at the Ser, Thr, and Tyr sites within the C-terminal helix modestly reduced the helicity and increased the turn/unordered regions without substantially altering the β-sheet population. The congruent effects of the pH titration and phosphorylation underscore a potentially shared electrostatic mechanism that fine-tunes Tau’s dynamic folding landscape. Although numerous studies have demonstrated that hyperphosphorylation accelerates fibrillation [68,69], the subtle perturbations observed here were measured in freshly prepared, monomeric pTau without prior incubation. This distinction underscores MMS’s ability to detect early, pre-aggregative structural changes that precede the extensive remodeling associated with fibril formation.

The MMS discriminated monomeric pTau from its heparin-induced aggregates by a quantitative increase in the β-sheet signature from ~30% in the native ensemble to ~50% in the fibrillar state, accompanied by a corresponding loss of turn/unordered content. Whether the transient β-strands detected in the native Tau act as obligatory precursors for amyloid formation remains unresolved. However, MMS now provides a high-throughput, label-free means to monitor this transition in real time and to interrogate how environmental or post-translational factors influence seeding competence, which can be extrapolated to studying Tau aggregation pathways in tauopathies such as Alzheimer’s Disease.

Despite its many advantages, MMS does have several important limitations that warrant careful consideration. MMS dependence on alternating sample-and-buffer flows for real-time background subtraction renders the method exceptionally sensitive to buffer mismatches; even subtle variations in ionic strength, pH, or excipient composition can induce subtraction artifacts that will compromise the amide I band fidelity. Samples containing high concentrations of chaotropes or salts (e.g., >4 M urea or >1 M NaCl) will produce disproportionately large solvent absorbance, exacerbating subtraction errors and rendering quantitation unreliable. Likewise, buffer components that engage in significant interactions or binding with the protein can alter their spectral signatures upon complex formation, leading to differential signals between samples and blanks and further mismatch risk. Consequently, rigorous buffer matching (via exhaustive dialysis, serial spin-column exchange, or use of identical preparative fractions) is essential, adding significant preparative overhead when screening multiple conditions or protein variants. Moreover, MMS only acquires ensemble transmission spectra, which are averaged, non-site-specific, and limited in reporting on tertiary contacts and distance constraints.

## 5. Conclusions

We have demonstrated that microfluidic modulation infrared spectroscopy provides a rapid method for resolving protein secondary structures, with exceptional sensitivity in native buffers. MMS accurately reproduces known fold compositions in globular proteins; can detect protein thermal transitions; and can report on subtle environment- and modification-driven perturbations in intrinsically disordered Tau, including pH- and phosphorylation-dependent shifts in α-helices, turn/unordered regions, and β-sheets. Critically, MMS distinguishes monomeric hyperphosphorylated Tau from its β-rich amyloid aggregates, quantifying the structural conversion underlying fibrillization. Collectively, these results establish MMS as a versatile platform for probing dynamic conformational ensembles and monitoring aggregation pathways, offering new avenues for mechanistic studies of protein folding, misfolding, and disease-associated assembly.

MMS’s minimal sample requirements and label-free operation further make it an ideal platform for high-throughput screening of modulators that stabilize or disrupt specific structural ensembles. Its ability to probe dynamic conformational landscapes under varied environmental and post-translational modifications positions MMS as a powerful tool for the future of drug discovery.

## Data Availability

All data is available upon request.
